# A COMPARATIVE ANALYSIS OF THE SOCIAL SUPPORT IN SOLITARY SENIORS WHO ATTEND CLUB ACTIVITY OR GO OUT TO SEE FRIENDS

**DOI:** 10.1093/geroni/igz038.628

**Published:** 2019-11-08

**Authors:** Mai Takase, Ryogo Ogino, Jun Goto, Junichiro Okata

**Affiliations:** 1 The University of Tokyo, Institute of Gerontology, Bunkyo-ku, Tokyo, Japan; 2 Institute of Gerontology, Tokyo, Japan; 3 Institute of Gerontology, The University of Tokyo, Tokyo, Japan; 4 The University of Tokyo, Institute of Gerontology, Tokyo, Japan, Japan

## Abstract

The Toyoshiki-dai housing complex, constructed in 1960s, is located in one of the commuter towns in a metropolitan area of Japan. Those who moved to this area as youngsters are now aged over 75 and many live a solitary life; making social support vital to prevent social frailty. The municipalities and universities have been hosting interventions, e.g. club activities, to make social connection but the effects remain unclear. In this study, the relationship between activity participation and the size of social support was explored to develop an effective method to increase social connections. A cross-sectional study was conducted in October 2018 and 200 questionnaires were distributed at a lunch event at the housing complex. The participants were solitary and independent seniors over the age of 70 (M:F=14.4:67.5). The frequency of going out to participate in club activity, see friends, and the geriatric social support scale were used for analysis. As a result, compared to seniors who went out to see their friends, the size of social support was smaller in groups that remained isolated (N = 161, odds ratio = 0.26, 95% CI=0.11-0.60). On the contrary, seniors who joined club activities had similar size of social support despite the frequency of participation. Clubs in Japan are often closed groups with limited membership, which may have restricted the addition of new social connection. Future intervention researches should focus on modifying the membership system of clubs and opening of a public space where seniors can casually access and talk to acquaintances.

